# Loss of the Cytoskeletal Protein Pdlim7 Predisposes Mice to Heart Defects and Hemostatic Dysfunction

**DOI:** 10.1371/journal.pone.0080809

**Published:** 2013-11-20

**Authors:** Jennifer Krcmery, Rajesh Gupta, Rudyard W. Sadleir, Molly J. Ahrens, Sol Misener, Christine Kamide, Philip Fitchev, Douglas W. Losordo, Susan E. Crawford, Hans-Georg Simon

**Affiliations:** 1 Department of Pediatrics, Feinberg School of Medicine, Northwestern University, Chicago, Illinois, United States of America; 2 Ann and Robert H. Lurie Children’s Hospital of Chicago Research Center, Chicago, Illinois, United States of America; 3 Feinberg Cardiovascular Research Institute, Feinberg School of Medicine, Northwestern University, Chicago, Illinois, United States of America; 4 Department of Pathology, Saint Louis University School of Medicine, St. Louis, Missouri, United States of America; Feinberg Cardiovascular Research Institute, Northwestern University, United States of America

## Abstract

The actin-associated protein Pdlim7 is essential for heart and fin development in zebrafish; however, the expression and function of this PDZ-LIM family member in the mammal has remained unclear. Here, we show that Pdlim7 predominantly localizes to actin-rich structures in mice including the heart, vascular smooth muscle, and platelets. To test the requirement for Pdlim7 in mammalian development and function, we analyzed a mouse strain with global genetic inactivation of Pdlim7. We demonstrate that Pdlim7 loss-of-function leads to significant postnatal mortality. Inactivation of Pdlim7 does not disrupt cardiac development, but causes mild cardiac dysfunction in adult mice. Adult *Pdlim7* ^*-/-*^ mice displayed increased mitral and tricuspid valve annulus to body weight ratios. These structural aberrations in *Pdlim7* ^*-/-*^ mice were supported by three-dimensional reconstructions of adult cardiac valves, which revealed increased surface area to volume ratios for the mitral and tricuspid valve leaflets. Unexpectedly, we found that loss of Pdlim7 triggers systemic venous and arterial thrombosis, leading to significant mortality shortly after birth in *Pdlim7* ^*+/-*^ (11/60) and *Pdlim7* ^*-/-*^ (19/35) mice. In line with a prothrombotic phenotype, adult *Pdlim7* ^*-/-*^ mice exhibit dramatically decreased tail bleed times compared to controls. These findings reveal a novel and unexpected function for Pdlim7 in maintaining proper hemostasis in neonatal and adult mice.

## Introduction

The PDZ-LIM family of proteins has been shown to regulate diverse biological functions, including bone morphogenesis, cardiac and skeletal muscle development and maintenance, neuronal signaling, and tumor cell growth [1,2]. Ten members comprise this protein family: Pdlim1-5, Pdlim7, LDB3, LMO7, and LIMK1 and LIMK2, sharing similar domain structures including one PDZ domain and one or more LIM domains. PDZ and LIM domains act as modular protein-binding interfaces to facilitate dynamic interactions with the actin cytoskeleton (α-actinin and β-tropomyosin), nuclear factors (Tbx4 and Tbx5), and signaling molecules (protein kinase C, ret/ptc2, and β1-integrin) [1,2]. Binding with multiple cofactors allows PDZ-LIM proteins to take on a variety of biological roles in different contexts. Importantly, several PDZ-LIM proteins have been found to function in cardiac and skeletal muscle development and maintenance in zebrafish and mice [3-9]. For example, mice lacking either Pdlim3, Pdlim5, or LDB3 develop dilated cardiomyopathy, and the latter knockout mice die within 5 days of birth due to severe striated muscle defects [4,5,8]. Pdlim1 is the only PDZ-LIM protein described to function in platelets [10], and loss of the protein in mice results in arterial thrombosis [11]. 

We have previously demonstrated that Pdlim7, which contains one PDZ and three LIM domains, associates with cytoskeletal actin [12], and dynamically regulates both the subcellular localization and activity of the nuclear transcription factor Tbx5 [13]. Further, knockdown of *pdlim7* in zebrafish results in loss of both cardiac valve tissue and pectoral fin outgrowth [3,14]. However, the functional importance of Pdlim7 in mammalian organogenesis has remained elusive. To gain insight into the biological significance of Pdlim7 in the mouse, we genetically inactivated the *Pdlim7* gene in all tissues. In contrast to the zebrafish, the global loss of Pdlim7 does not disrupt mouse cardiac development, but causes mild cardiac dysfunction and valve structural defects in adults. Interestingly, loss of or reduced levels of Pdlim7 in homo- and heterozygous mutant mice, respectively also results in systemic, occlusive thrombosis leading to significant early lethality with survivors displaying decreased tail bleed times. These findings reveal an unexpected and previously unknown *in vivo* function for Pdlim7 in maintaining hemostasis. 

## Materials and Methods

### Global deletion of the Pdlim7 gene in ES cells by retroviral insertion

*Pdlim7* mutant mice were generated by Lexicon Genetics Inc. from 129Sv/Ev embryonic stem (ES) cells (OST445990) using a gene trap approach as previously described [15,16]. *Pdlim7* was disrupted by insertion of the VICTR37 gene trap vector in ES cells: this allele is therefore named *Pdlim7* ^*Gt(VICTR37)445990Lex*^. For simplicity, we will refer to *Pdlim7* ^*+/Gt(VICTR37)445990Lex*^ mice as *Pdlim7* ^*+/-*^ and *Pdlim7* ^*Gt(VICTR37)445990Lex/ Gt(VICTR37)445990Lex*^ as *Pdlim7* ^*-/-*^. Direct sequencing of products from 3’ rapid amplification of cDNA ends (RACE) determined the integration site to be in intron 2 of the *Pdlim7* gene. *Pdlim7* ^*+/-*^ hybrid mice were backcrossed 5 generations onto the C57BL/6 background. *Pdlim7* mutant mice were genotyped using multiplex PCR analysis of tail genomic DNA using the following primers to detect mutant and WT Pdlim7: (WT Pdlim7 forward primer) 5’ACCAGCTTAGCCCTCACATTT3’: (WT Pdlim7 reverse primer) 5’TACGTGTGATGCTAACACTCAGGC3’: (viral LTR2 reverse primer) 5’ATAAACCCTCTTGCAGTTGCATC3’. All protocols involving animals in this work were approved by the Institutional Animal Care and Use Committee of Northwestern University and the Ann and Robert H. Lurie Children’s Hospital of Chicago Research Center. 

### Semi-quantitative RT-PCR expression analysis

Total RNA from adult uteri was prepared using the NucleoSpin RNAII kit including DNase treatment (Clontech, Mountain View, CA) as previously described [12]. RNA from the human and murine megakaryocyte cell lines, K562 and Y10, respectively, were a kind gift from Dr. John Crispino. Total RNA from washed mouse platelets was prepared as previously described by Rowley et al [17]. Oligonucleotides specific for the individual genes were: Human GAPDH: (FWD) 5’-CGTCATGGGTGTGAACCATGAGAA, (REV) 5’-GCC AGT AGA GGC AGG GAT GAT GTT; Mouse GAPDH: (FWD) 5’-TGTGATGGGTGTGAACCACGAGAA, (REV) 5’- ACCAGTGGATGCAGGGATGATGTT; Human Pdlim7: (FWD) 5’-AACAACGGCAAGACTCCCGTGTGT, (REV) 5’-TCTTGCACTTGGCACAGCTGGGTG; Mouse Pdlim7: (FWD) 5’-AACAACGGCAAGACTCCTGTATGC, (REV) 5’-TCTTGCATTTGGCACAGTTGGGTG.

### Quantitative RT-PCR expression analysis

Quantitative RT-PCR was performed on adult uteri RNA (see above) using pre-validated Solaris gene specific primers *Pdlim7* and *GAPDH* (Thermo Scientific) on the Step One Plus Real-Time PCR System (Applied Biosystems) using the Solaris qPCR ROX Master Mix (Thermo Fisher) for detection of amplified DNA. Data was analyzed using the comparative ∆∆Ct method and normalized to *GAPDH* control. Normalized values were converted to relative values by using the corresponding WT uteri as the calibrator. 

### Western-blot analysis

Total protein from adult uteri and platelets (see below for platelet isolation protocol) was obtained by homogenization in RIPA buffer containing Halt Protease Inhibitor and Halt^TM^ Phosphatase Inhibitor Cocktails (Thermo Scientific) according to manufacturer protocol. The homogenate was centrifuged at 16,000 x g for 20 minutes at 4°C and supernatant containing the protein removed. Protein concentrations were determined by a BCA assay (Pierce Biotechnology) for subsequent SDS-PAGE and immunoblot analysis using a BioRad ChemiDoc MP system with anti-Pdlim7 [13] and anti-GAPDH (Santa Cruz) antibodies followed by measurement of relative densitometry. 

### Beta-galactosidase staining

*Pdlim7* mutant embryos from E9.5-18.5 were fixed with 4% paraformaldehyde and for sections, embedded in OCT medium and processed for cryosectioning on a Leica CM3050S cryostat (Leica Microsystems). Whole-mount embryos and sections were stained with 1 mg/ml X-gal overnight at 37°C as previously described [18,19]. Sections were briefly counter-stained with nuclear fast red to visualize overall morphology. Whole-mount embryos were imaged on a Leica MZ16 stereomicroscope fitted with a Leica DFC490 color camera using ImagePro MC (Media Cybernetics) software. For these and all subsequent colorimetric stains, sections were imaged on a Leica DMR upright microscope equipped with a QImaging Retiga 4000R camera using OpenLab (Improvision) software. All images were processed using Photoshop CS4 (Adobe Systems, Inc). 

### Immunofluorescence

Indirect immunofluorescence detection was performed as previously described [13,20]. In short, tissue cryosections or washed platelets (see below for isolation protocol) were fixed with 4% paraformaldehyde, permeabilized with 0.5% Triton X-100, and blocked with 20% goat serum, 5% 20X blocking solution (50mM NH_4_Cl, 25mM Lysine, and 25mM Glycine), and 0.2% BSA in PBS. Tissues or platelets were incubated with primary antibodies including, anti-Pdlim7 [13], anti-PECAM (BD Biosciences) and anti-MF20 (Developed by D.A. Fischman, Developmental Studies Hybridoma Bank, University of Iowa) antibodies and detected using Alexa 488- or 564-conjugated secondary antibodies (Invitrogen). Filamentous actin and nuclei were detected using Alexa Fluor 546 or 633 (Invitrogen) and DAPI (Roche), respectively. Fluorescence staining was visualized on a Zeiss LSM510 META confocal microscope. 

### Echocardiography

Transthoracic echocardiography was performed using the VisualSonics 770 Imaging System (Toronto, Canada) equipped with a 30-mHz transducer. Mice were anesthetized with 1-2% isoflurane inhalation with heart rate (360-500 beats per minute) and core temperature (36-37°C) continuously monitored. Annulus dimensions were obtained in the apical 4-chamber view during end diastole with mitral and tricuspid valves in the closed position. Pulsed wave doppler interrogation was performed on the mitral valve inflow in the apical 4-chamber view using a sample volume toggle to optimally assess flow velocities. All measurements were obtained using an angle of interrogation <30° [21]. The isovolumetric relaxation time (IVRT), isovolumetric contraction time (IVCT), and ejection time (ET) were also measured from the mitral inflow doppler signal [22-24]. The Myocardial Performance Index (MPI) or Tei index was calculated as follows: (IVRT + IVCT) / ET. Higher values in the MPI indicate worsening cardiac function. Cardiac chamber dimensions, wall thickness, and left ventricular cardiac function (ejection fraction and fractional shortening) were measured from images obtained using two-dimensional M-mode echocardiography in the parasternal long and short axis views [25]. All measurements were obtained in triplicate and averaged.

### Histological Analysis

Specimens were fixed in 4% paraformaldehyde, dehydrated, embedded in paraplast, and sectioned on a Leica RM2265 microtome (Leica Microsystems). For the embryonic and perinatal studies, sections were stained with 1% Alcian blue pH2.5/nuclear fast red and Hematoxylin & Eosin, respectively.

For the three-dimensional morphometric analysis, 3-month old hearts were first heparinized, relaxed, and perfused prior to fixation [25] to obtain better visualization of valve tissue. Specifically, 50 µL of 1,000 U/mL heparin was injected directly into the right ventricle and allowed to circulate to prevent clotting of the blood. The abdominal aorta and descending vena cava were then cut for fluid overflow. Next, 500 µL of relaxin solution was injected into each ventricle to relax the heart and keep the atrioventricular valves open, which was followed by perfusion with a total of 5 mL of PBS. The heart was then removed from the chest cavity and a 22G teflon catheter inserted into the aorta (past the aortic and mitral valves) followed by perfusion with 5mL PBS. Hearts were serial sectioned and stained with either 1% Alcian Blue pH2.5/nuclear fast red or Masson’s Trichrome staining. 

### Three-dimensional morphometric analysis

Adult heart serial sections were imported into Amira v5.4.0 (Visage Imaging) at a scaled unit pixel size of 1x1x1.285 to derive the comparative valve metrics. Sections were manually aligned, followed by a least squares computed alignment to minimize the number of non-overlapping pixels between two sequential images. The AV valve tissue was manually segmented with the aid of a threshold mask on a gray-scale image series by referencing the color images for each heart section. For the surface area and volume computation, a triangulated surface was computed from the voxel segmentation using the constrained smoothing algorithm option. The surface area and volume of the AV valves were computed using Amira’s Surface Area module. 

### Blood collection and counts

Blood was drawn by cardiac puncture from adult mice anesthetized with 2-3% isoflurane inhalation into either heparin or 3.2% sodium citrate at a ratio of 1:9. Blood cell counts were performed on a Beckman Coulter AcTdiff2 Analyzer. To obtain washed platelets, whole blood was centrifuged at 86 x g for 8 minutes to collect platelet-rich plasma (PRP). To increase platelet yield, the lower phase was washed 3 times with HEPES/Tyrode’s buffer and the obtained PRP were combined. The PRP was then spun in the presence of prostaglandin I2 (0.1 µg/mL) at 718 x g for 6 minutes. The platelet pellet was washed in HEPES/Tyrodes buffer pH7.4 (10mM HEPES, 12mM NaHCO_3_, 138mM NaCl, 5.5mM glucose, and 2.9mM KCl) [26] and either re-pelleted for Western blot or resuspended in HEPES/Tyrodes buffer pH7.4 containing 2 U/mL apyrase and 10µm indomethacin at a concentration of 50,000 plts/µL and allowed to spread on glass coverslips for 5 minutes at 37°C followed by indirect fluorescence immunostaining as described above. Platelet-poor plasma was obtained by centrifugation of whole blood at 2000 x g for 10 minutes. The liver chemistries, prothrombin, and partial thromboplastin times were performed by Antech Diagnostics (Oak Brook, IL). 

### Tail bleeding time assay

The tail bleeding time assay was carried out with the operator blinded to the genotype of the mice. Weaning age mice were sedated with Acepromazine (2mg/kg) and placed on a raised platform with tails protruding over the edge. Tails were positioned 5mm above filter paper and a 2-3mm cut was made at the distal tip of the tail with a scalpel. The bleeding time was defined as the time needed for the cessation of bleeding (no blood drops for 1 minute) [27]. 

### Statistics

All values are expressed as mean SD. Comparisons between two groups were evaluated with the unpaired *t* test and between three groups with one-way ANOVA. The Kruskal-Wallis one-way analysis of variance was used to evaluate body weights, bleeding time, and the following blood cell and chemistry counts: monocyte, granulocyte, aspartate aminotransferase (AST), and total bilirubin, which were non-normally distributed. P<0.05 was considered statistically significant.

## Results

### Pdlim7 is dynamically expressed in actin-rich structures during murine development

**Figure 1:**
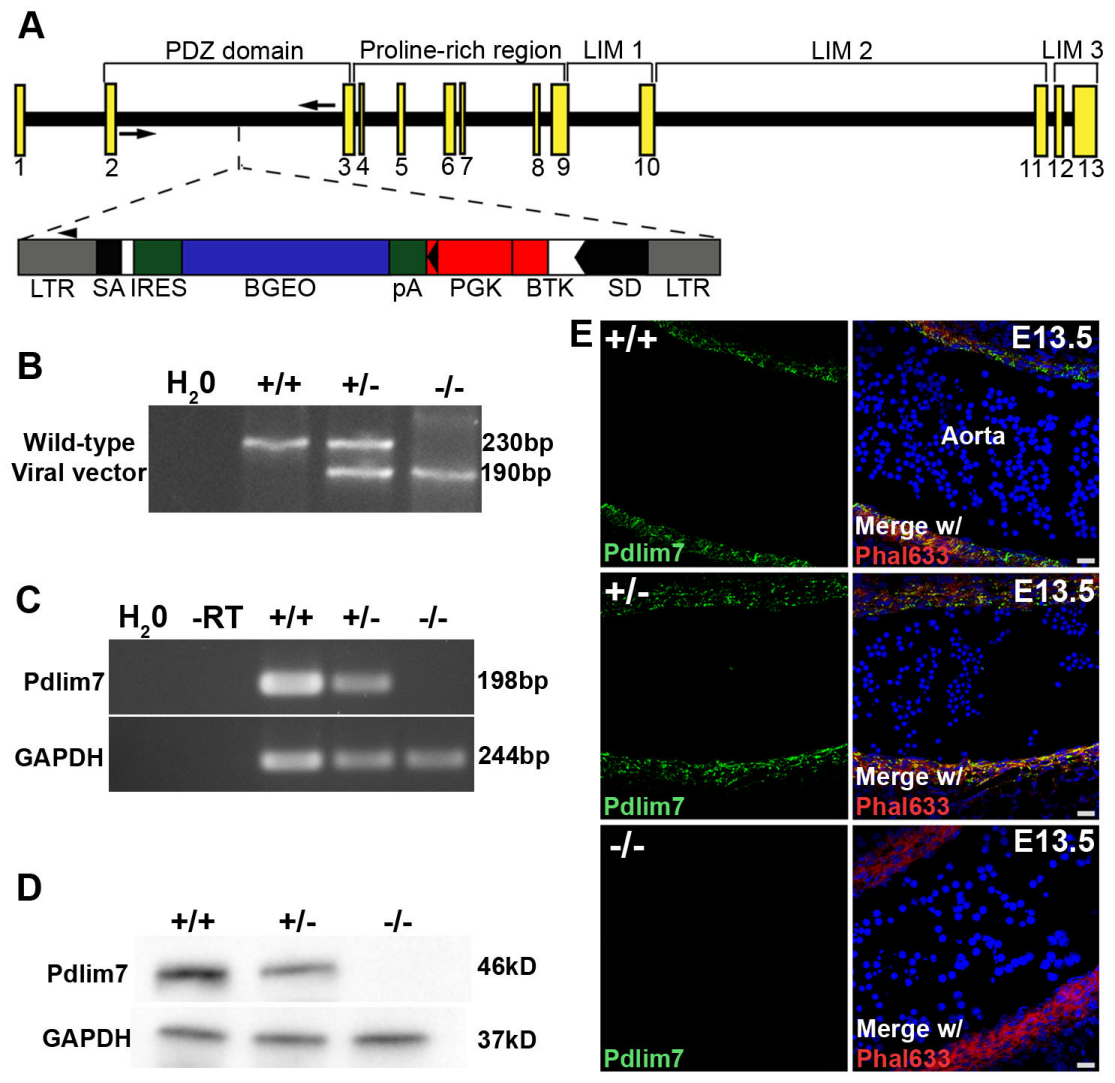
Generation of *Pdlim7* mutant mice. Schematic representation of the retroviral integration site within intron two of the *Pdlim7* gene (A). Exons are depicted by yellow boxes with WT and viral-specific genotyping primers represented by arrows and an arrowhead, respectively. Genotyping reveals a 230bp PCR fragment representing the WT allele and a 190bp product for the viral integration (B). Semi-quantitative RT-PCR demonstrates absence of *Pdlim7* gene transcripts in adult uteri of Pdlim7^-/-^ mice using GAPDH as control (C). Western blot shows that Pdlim7^-/-^ and Pdlim7^+/-^ mice express only -1.18 ± 2.64% and 29.36 ± 14.92% Pdlim7 protein compared to WT controls, respectively (D). Sagittal section through the aorta demonstrates Pdlim7 antibodies (green) localize to filamentous actin (red) of smooth muscle in WT, but not Pdlim7^-/-^ embryos (E), further verifying loss of Pdlim7 protein in null mice (control DAPI nuclei, blue). LTR = long terminal repeat; SA and SD = splice acceptor and donor sites, respectively; IRES = internal ribosomal entry site; BGEO = lacZ gene; pA = polyadenylation signal; PGK = phosphoglycerate kinase gene; BTK = Bruton’s tyrosine kinase gene.

In order to study the functional role of Pdlim7 in the mammal, we chose a retrovirus insertion into intron two of the mouse *Pdlim7* gene (Figure 1A-B), as this disruption is expected to globally abolish protein expression. Loss of *Pdlim7* mRNA expression was verified in adult *Pdlim7* mutant uteri using semi-quantitative RT-PCR (Figure 1C). The reduction in mRNA was further verified by quantitative RT-PCR, revealing a decrease in *Pdlim7* transcript levels in the respective uteri of *Pdlim7* ^*+/-*^ and *Pdlim7* ^*-/-*^ mice of 61.27 15.25% and 99.37 0.06%, respectively (n≥3). Additionally, Western-blots with anti-Pdlim7 antiserum [13] recognized Pdlim7 protein in the smooth muscle-rich uterus of wild-type (WT) adult mice; however, protein expression in the respective uteri of *Pdlim7* ^*+/-*^ and *Pdlim7* ^*-/-*^ mice was only 29.36 14.92% and -1.18 2.64% of protein levels in WT mice (Figure 1D; n=5). In agreement with the Western-blot, immunohistochemistry with anti-Pdlim7 antiserum revealed Pdlim7 protein in the aorta of WT embryos while protein expression in the respective aorta of *Pdlim7* ^*-/-*^ littermates could not be detected (Figure 1E). Thus, we conclude that the *Pdlim7* allele generated is a null allele.

**Figure 2:**
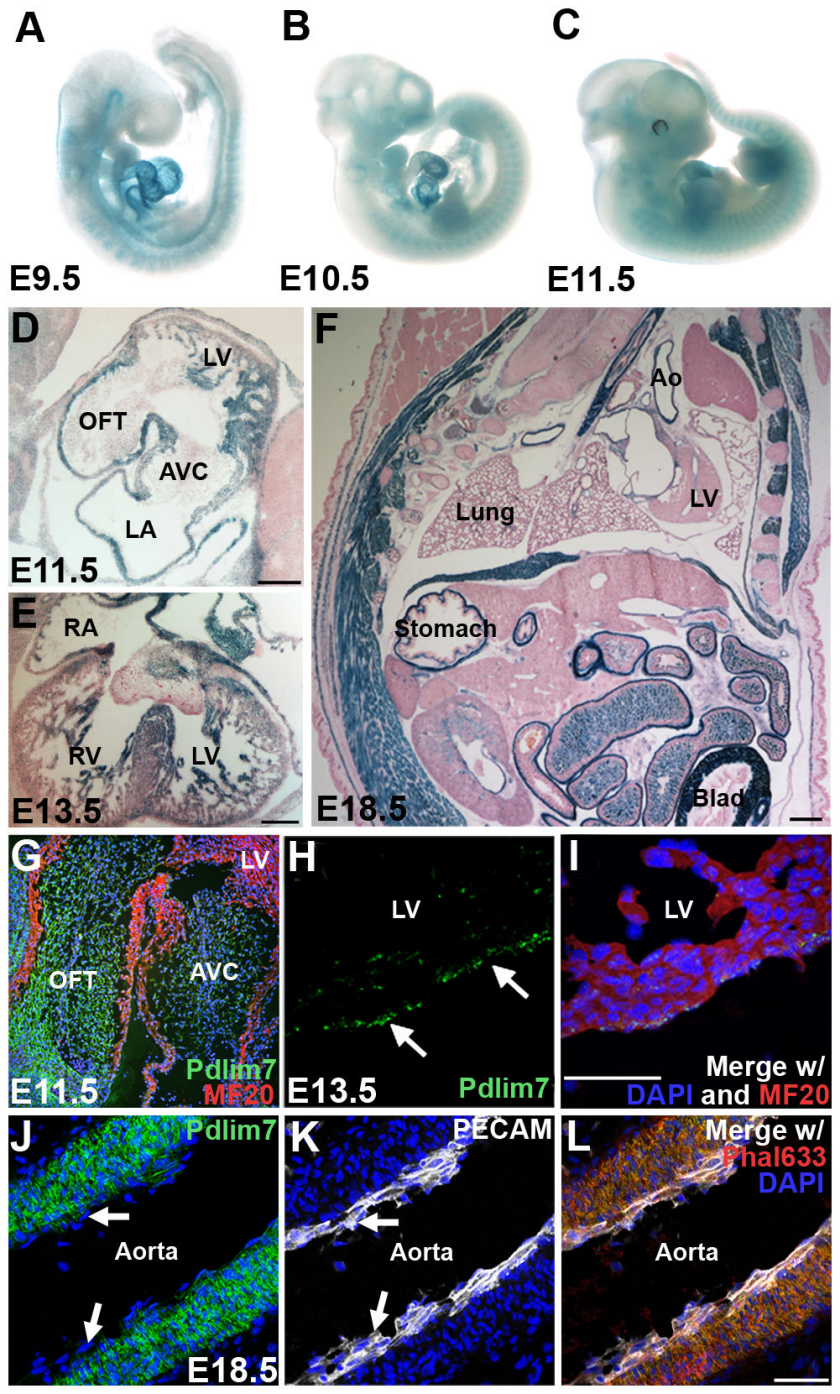
Pdlim7 is dynamically expressed in actin-rich structures throughout murine development. β-galactosidase staining of whole mount embryos identifies *Pdlim7* early in the developing heart, forelimbs, hindlimbs, and somites (A-C). In E11.5 and E13.5 heart sections, β-galactosidase staining is localized to the trabeculated regions of the ventricles, atrial walls, and interventricular and atrial septa (D-E); of note: the β-galactosidase reaction time chosen does not reveal the lower levels of Pdlim7 expression in the cardiac cushion or valve cells. Sagittal sections of E18.5 embryos show Pdlim7 expression throughout the smooth muscle, including the lung alveoli, stomach, aorta, and bladder (F). Immunostaining of E11.5 hearts demonstrates Pdlim7 (green) localization to the outflow tract and atrioventricular cushions with MF20 positive myocardial cells (red) (G). Pdlim7 (green) proteins are also detected in the epicardium of E13.5 embryos (arrows, H-I) with MF20 positive myocardial cells (red) and DAPI nuclei (blue). In the aorta, Pdlim7 proteins (green) localize to filamentous actin (red) of smooth muscle, but not PECAM-positive endothelium (white) (arrows, J-L), control DAPI nuclei (blue). Scale bar = 200μm (D-E) and 50 μm (F, I, L). Ao = Aorta; AVC = atrioventricular cushion; Blad = bladder; LA = left atrium; LV = left ventricle; OFT = outflow tract; RA = right atrium; RV = right ventricle.

Taking advantage of the gene trap containing a *lacZ* reporter in frame with *Pdlim7* exon coding sequences (Figure 1A), we used β-galactosidase staining to establish a previously unknown expression profile of *Pdlim7* in the mouse. In accordance with our studies in the zebrafish [14] and chicken [12,20], murine *Pdlim7* was dynamically expressed throughout embryogenesis, particularly in the developing somites, forelimbs and hindlimbs, and heart (Figure 2A-C). We detected robust expression of *Pdlim7* at E9.5 through E10.5 in a rostral to caudal gradient in the somites (Figure 2A-B) and at E11.5 in cells migrating from the myotome (Figure 2C). At E18.5, *Pdlim7* was also detected throughout the embryonic smooth muscle including the stomach, bladder, duodenum, lungs, esophagus, and aorta (Figure 2F and Table S1 in Methods S1). In the developing heart, we detected *Pdlim7* transcripts in the atria, trabeculated regions of the ventricles, and the interventricular and atrial septa (Figure 2D-E and Table S1 in Methods S1).

To obtain more detailed information on Pdlim7 protein distribution, we utilized immunohistochemistry with our anti-Pdlim7 antiserum [13], which is cross-reactive in mouse tissue (Figure 1E). We noted from NCBI searches that Pdlim7, like other PDZ-LIM proteins, is expressed in multiple splice variants (data not shown). Our Pdlim7 antibodies, however, were designed to exclusively detect the full-length protein composed of a PDZ domain, proline-rich region, and three LIM domains and, thus, was not expected in all cases to produce an overlapping pattern with the β-galactosidase staining. For example, we found *Pdlim7* transcripts but no antibody reaction in the left atrium and trabeculated regions of the left ventricle of E11.5 embryos (Figure 2D and G), suggesting that these domains express shorter splice forms of the *Pdlim7* gene. Consistent with protein distribution in the chicken [20], our immunostaining detected Pdlim7 proteins in the developing atrioventricular (AV) and outflow tract (OFT) cushions of the heart as well as the epicardium (Figure 2G-I). This more sensitive technique detected expression that was not readily apparent from β-galactosidase staining. We also confirmed Pdlim7’s selectivity to smooth muscle by performing immunostaining with anti-Pdlim7 and anti-platelet endothelial cell adhesion molecule (PECAM)-1 antibodies along with an actin stain (Phalloidin). We found Pdlim7 co-localized with filamentous (F)-actin in aortic smooth muscle, but not in the endothelial layer (arrows, Figure 2J-L). Overall, these expression data revealed that Pdlim7 predominantly localizes to actin-rich structures throughout mammalian development. 

### Non-Mendelian ratios are observed in Pdlim7 mutant mice

From *Pdlim7* heterozygous crosses, we noticed that the percentage of *Pdlim7* ^*+/+*^ (23.2), Pdlim7^+/-^ (39.2), and *Pdlim7* ^*-/-*^ (12.8) were not obtained in the expected 25:50:25 Mendelian ratios at weaning (Table 1; n=94; Chi square test p=0.0006). Close observation revealed that non-surviving Pdlim7^+/-^ (11/60) and *Pdlim7* ^*-/-*^ (19/35) pups were born alive, but died within 48 hours of birth while the survivors lived over 1 year of age. Notably, the percent postnatal lethality strongly correlates with *Pdlim7* ^*-/-*^ and *Pdlim7* ^*+/-*^ mice expressing only -1.18 2.64% and 29.36 14.92% of protein levels in WT mice, respectively (Figure 1D). 

**Table 1:** Mendelian ratios are not obtained at weaning from *Pdlim7* heterozygous crosses.

**Age**	**N-value**	**Pdlim7^+/+^ (%)**	**Pdlim7^+/-^ (%)**	**Pdlim7^-/-^ (%)**
Birth	125	24.0	48.0	28.0
P21	94	23.2	39.2	12.8

Gross assessment of *Pdlim7* mutant mice revealed that at birth, *Pdlim7* ^*-/-*^ pups (n=3) had comparable body weights to WT littermates (n=7; 1.34 0.11 g vs. 1.27 0.12 g, p=0.3608); however, at 3-months of age, *Pdlim7* ^*-/-*^ mice (n=8; 22.4 0.5 g) exhibited a reduction in body weight compared to WT littermates (n=13; 26.3 2.3 g) with the *Pdlim7* ^*+/-*^ mice (n=13; 25.1 1.8 g, p=0.0003) displaying an intermediate phenotype.

### Pdlim7-deficient mice maintain normal cardiac development while adults exhibit mild cardiac dysfunction and aberrant cardiac valve shape

Knockdown of *pdlim7* in zebrafish causes improper AV boundary formation, leading to loss of the cardiac chamber valve [14]. Therefore, we hypothesized that a primary cardiac anomaly, especially related to the AV valves, may contribute to the lethality in *Pdlim7* mutant mice. In mice, valve development begins at the AV junction where specialized myocardial cells secrete growth factors of the TGF gene family (TGFβ1-3, BMP2 and 4) that induce endocardial epithelial-to-mesenchymal transition (EMT) and promote endocardial cushion formation [28-33]. The endocardial cushions undergo complex remodeling whereby the cushion mesenchyme differentiates into fibroblastic interstitial cells characteristic of mature valves [34-38]. 

 To our surprise, cardiac valve development in *Pdlim7* ^*-/-*^ mice appeared normal. A multi-stage histological analysis of Pdlim7^-/-^ embryonic hearts with a focus on the endocardial cushions and developing valves did not reveal significant morphological abnormalities compared to WT controls (Figure S1; n>3; Materials S1). To assess at the cellular level whether endocardial EMT was abnormal in Pdlim7-deficient mice, we utilized an ex vivo AV cushion explant assay [39]. We were unable to detect significant differences in the transformation of epithelial-to-mesenchymal cells and subsequent invasion into the collagen gel between *Pdlim7* ^*-/-*^ embryos and WT controls (Figure S2A-D; n=3; Materials S1). In addition to our histological and cellular assessment of EMT progression, we performed molecular analyses. Utilizing whole-mount *in situ* hybridization, we found the spatiotemporal expression of *Bmp2*, a key regulator of endocardial EMT [40,41], to be relatively normal in *Pdlim7* ^*-/-*^ embryos during critical stages (E9.5 and E10.5) of cushion formation (Figure S2E-H; n>3; Materials S1). A gene pathway PCR array selected for genes involved in EMT further verified that genes known to be important for endocardial cushion development, (e.g. *Erbb3*, *Notch1, Snai1*, *Tgfb2*, *Twist1*, and *Versican* (*Vcan*)), including several downstream of Bmp2, remained unchanged following loss of Pdlim7 in mice (Figure S2I; <2-fold differential expression; Materials S1). Taken together, these findings indicate that in mice, Pdlim7 does not appear to be essential for early cardiac AV valve formation. 

**Figure 3:**
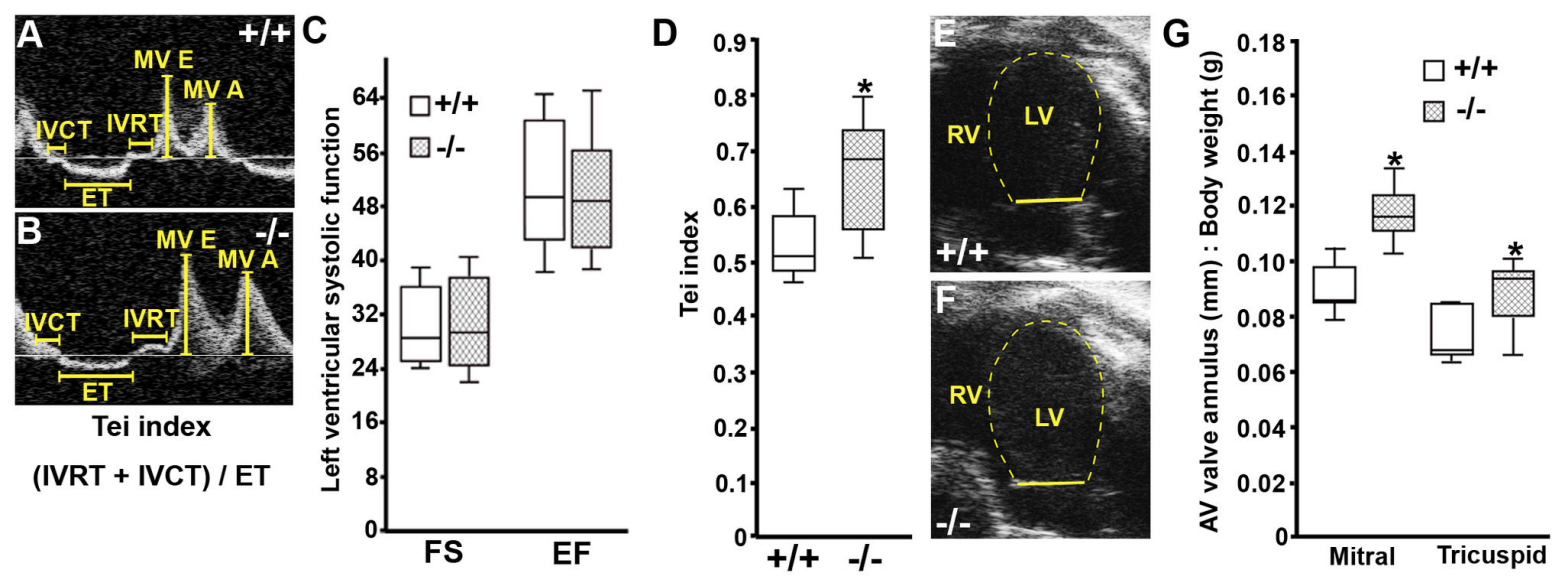
Adult Pdlim7^-/-^ mice exhibit mild cardiac dysfunction with increased valve annulus dimensions. Representative Doppler profile in the apical-4 chamber view of mitral valve inflow velocities (A-B) and mitral valve annulus measurement (E-F) from WT (n=8) and Pdlim7^-/-^ (n=9) mice. Box-and-whisker plots reveal normal fractional shortening (FS) and ejection fraction (EF) in Pdlim7^-/-^ mice (C), but an increased Tei index (D) and increased mitral and tricuspid annulus dimensions to body weight ratios (G) as compared to controls, *p<0.01. A = late filling; AV = atrioventricular; E = early filling; ET = ejection time; IVCT = isovolumetric contraction time; IVRT = isovolumetric relaxation time; LV = left ventricle; MV = mitral valve; RV = right ventricle.

Several PDZ-LIM protein loss-of-function studies in mice indicated later onset cardiac defects [5,8], especially compared to similar analyses done in zebrafish [6]. Thus, we sought to determine whether Pdlim7 loss-of-function leads to later onset cardiac defects. Doppler transthoracic echocardiography on 3-month old *Pdlim7* ^*-/-*^ (n=9) and WT mice (n=8), however, did not reveal significant differences in left ventricular output during the contracted phase (systole) of the cardiac cycle as measured by ejection fraction (EF) and fractional shortening (FS) (Figure 3C, Table S2 in Methods S1; p>0.05). We did find that adult Pdlim7^-/-^ mice displayed an elevated myocardial performance index or Tei index (Figure 3A-B and D, Table S3 in Methods S1; 0.66 ± 0.09 vs. 0.53 ± 0.06, p=0.0036), which assesses cardiac function during both the relaxed and contracted phases of the cardiac cycle [24,42,43], indicating mild cardiac dysfunction. Additionally, we found that adult Pdlim7^-/-^ mice (n=9) had increased mitral (0.12 ± 0.01 mm/g) and tricuspid (0.09 ± 0.01 mm/g) annulus diameter to body weight ratios in comparison to WT (Figure 3E-G; n=8, 0.09 ± 0.01 and 0.07 ± 0.01 mm/g, respectively p=0.000018 and 0.0096). Left ventricular wall thickness and inner chamber dimensions during the cardiac cycle remained unchanged (Table S2 in Methods S1; n≥8, p>0.05), suggesting that the increased annulus was not a result of an enlarged chamber, but rather a primary malformation related to valve size.

**Figure 4:**
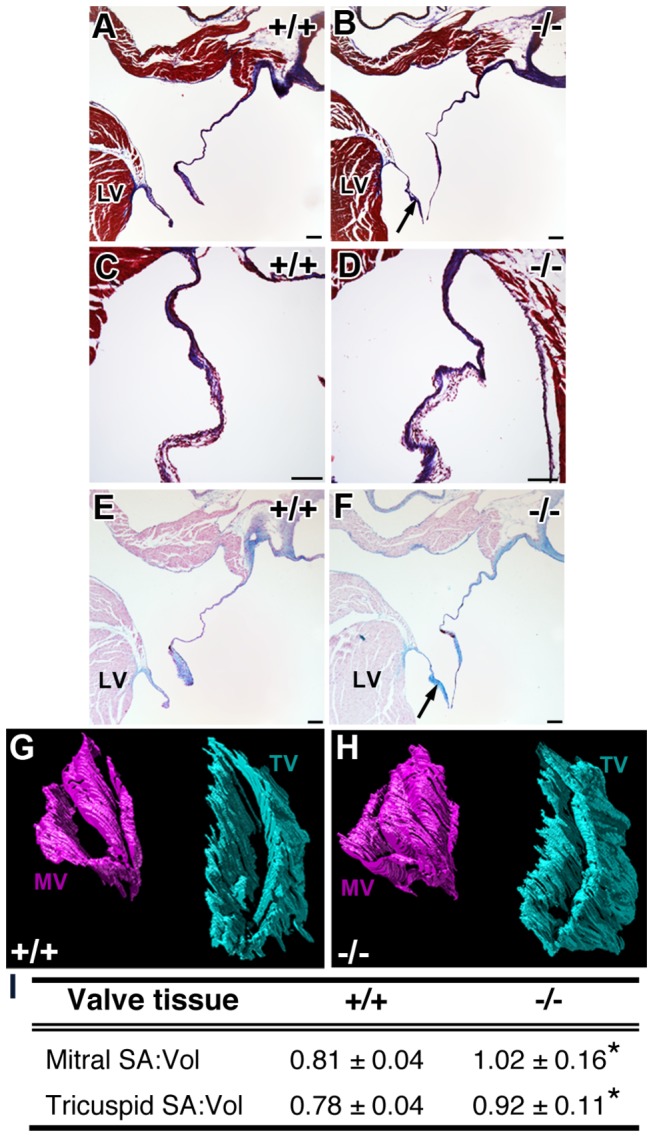
Atrioventricular valves of adult Pdlim7^-/-^ mice display abnormal morphology. Histological sections from 3-month old WT and Pdlim7^-/-^ hearts stained with Masson’s Trichrome (A-D) and Alcian blue (E-F) exhibit normal distribution of collagens (blue, A-D) and glycosaminoglycans (blue, E-F), but elongated mitral valves in Pdlim7^-/-^ mice (arrows, B, F; n=5) compared to WT controls (A, E; n=3). Scale bar = 100 μm. Representative 3D reconstructions of serial sections of mitral (pink) and tricuspid (turquoise) valves from a WT (G) and Pdlim7^-/-^ (H) mouse in the same anterodorsolateral perspective. Table providing quantification of increased surface area to volume ratios of atrioventricular valves in Pdlim7^-/-^ (mitral, n=5; tricuspid, n=4) compared to WT (mitral, n=6; tricuspid, n=5) controls (I). *p<0.04. LV = left ventricle; MV = mitral valve; SA = surface area; TV = tricuspid valve; Vol = volume.

Following echocardiography, the 3-month old hearts were subjected to histological analysis in multiple orientations. Masson’s trichrome and Alcian blue staining revealed normal distribution of collagens and glycosaminoglycans in AV valve sections, respectively, but an elongation of the leaflets in *Pdlim7* ^*-/-*^ hearts (arrows, Figure 4A-F). In order to reliably measure valve shape and size, we utilized AMIRA software to assemble a complete series of adjacent histological sections in both coronal and transverse orientations into 3-dimensional (3D) valve models. Using surface area to volume (SA:Vol) ratios as a measure of shape and size [44], we quantified the difference between *Pdlim7* ^*-/-*^ and WT valve leaflets. We determined that *Pdlim7* ^*-/-*^ mice have increased SA:Vol ratios for both the mitral (n=5; 1.015 0.157) and tricuspid (n=4; 0.917 0.111) valves in comparison to WT (Figure 4G-I and Video S1; n=6, 0.809 0.043, p=0.0127 and n=5, 0.782 0.038, p=0.0363, respectively). The increased SA:Vol ratio translates into longer, thinner valves, suggesting an abnormal remodeling of the AV valves in the absence of Pdlim7. 

### Occlusive vascular thrombosis leads to early mortality in Pdlim7 mutant mice

**Figure 5:**
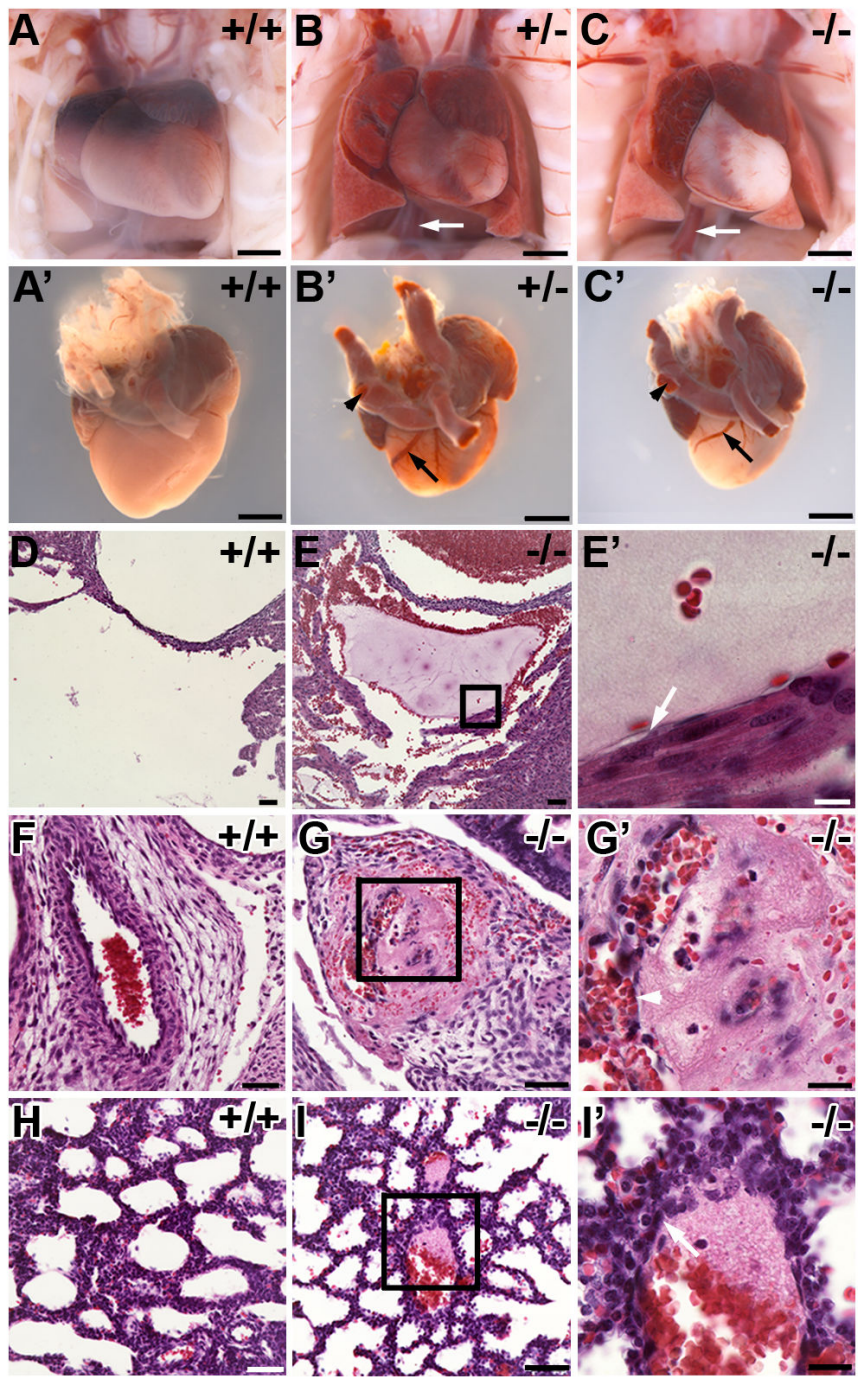
Pathological analysis of non-surviving *Pdlim7* mutant pups reveals pre-mortem thrombi. *Pdlim7* ^+/-^ (n=3) and *Pdlim7* ^-/-^ (n=11) perinatal lethal pups exhibit extensive blood clots in the heart, arteries (arrowhead), and veins (arrows, B-C’) associated with atrial dilation and lung congestion compared to WT (n=7) controls (A-A’). Example of pre-mortem blood clots in the right ventricle (E-E’), umbilical vessel (G-G’), and lung alveoli (I-I’) of a Pdlim7^-/-^ perinatal lethal pup compared to WT control (D, F, H). Boxes depict location of high magnification image in E’, G’, and I’. Arrows point to attachment of the thrombus to the vessel wall (E’, I’) and arrowhead points to recanalization of the clot (G’). Scale bar = 1mm (A-C’), 50 µm (D-I), 20 µm (G’, I’), and 10µm (E’).

Unexpectedly, while examining adult *Pdlim7* ^*-/-*^ hearts prior to perfusion, we noticed, in some cases, blood clots attached to the AV valves (Figure S3; Materials S1). These findings prompted an examination of a clotting issue that may explain the early mortality in *Pdlim7* mutant mice. Gross morphological assessment of the non-surviving Pdlim7^+/-^ (n=3) and Pdlim7^-/-^ (n=9) pups revealed extensive vascular thrombi associated with dilatation of the atria and coronary vasculature as well as lung congestion (Figure 5B-C’). Histological analysis confirmed the presence of pre-mortem occlusive venous and arterial thrombi in several organ systems of non-surviving Pdlim7^+/-^ (n=7) and Pdlim7^-/-^ (n=6) pups, with each pup exhibiting multiple thrombi in more than one organ (Figure 5 and Table 2). For example, note the mural thrombus in the right ventricle of a *Pdlim7* ^*-/-*^ heart (Figure 5E-E’) and the organized thrombus in the umbilical vessel with evidence of recanalization (Figure 5G-G’). We also found early organizing thrombi in the arterioles of lungs in almost all of the non-surviving Pdlim7^-/-^ pups (5/6; Figure 5I-I’) with additional clots in the hepatic vein (4/6) and coronary vessels (4/6) (Table 2). Of note, we did not observe organizing thrombi in *Pdlim7* ^*-/-*^ mice prior to birth (data not shown), indicating that the clots were forming after birth. These findings indicate that a deficiency in Pdlim7 results in systemic, pre-mortem vascular thrombosis leading to significant mortality. 

**Table 2:** Location and prevalence of pre-mortem thrombi in *Pdlim7* mutant perinatal lethal pups.

**Cardiovascular System**	**Pdlim7^+/-^**	**Pdlim7^-/-^**
Aorta	2/7	2/6
Coronary vessels	4/7	4/6
IVC / SVC	1/7	2/6
Atria	3/7	3/6
Ventricles	1/7	2/6
AV valves	3/7	2/6
**Pulmonary System**		
Lung alveoli	7/7	5/6
Pulmonary artery	0/7	1/6

AV = atrioventricular; IVC = inferior vena cava; SVC = superior vena cava.

### Pdlim7 mutant survivors exhibit hemostatic dysfunction

Based on the neonatal findings and blood clots on the AV valves of Pdlim7^-/-^ adult mice, we sought to further characterize a role for Pdlim7 in regulating adult hemostasis. To better understand the etiology of the hemostatic dysfunction, we performed a range of blood tests to assess any changes following loss of Pdlim7. Prothrombotic phenotypes in mice have almost exclusively been linked to deficiencies in plasma proteins [45-47], thus we also tested whether the plasma proteins related to the extrinsic (prothrombin time, PT) and intrinsic (partial thromboplastin time, PTT) coagulation pathways were affected. Both the PT and PTT assays revealed no significant difference between *Pdlim7* ^*-/-*^ (PT and PTT n=6) and WT mice (Table 3; PT n=8 and PTT n=4; p>0.05), indicating that the coagulation pathways function normally in the absence of Pdlim7. The liver synthesizes clotting factors, coagulation inhibitors, and fibrinolytic proteins [48], but its central role in hemostasis is also emphasized by liver diseases that are accompanied by hemostatic changes, leading to either bleeding or thrombosis [48,49]. We conducted a liver chemistry panel, but did not find significant differences between *Pdlim7* ^*-/-*^ (n=6) and WT (n=6; p>0.05) mice for any of the parameters tested, such as total bilirubin, albumin, aspartate aminotransferase (AST), or alanine aminotransferase (ALT) (Table 3). In addition to the liver chemistries, we performed a complete blood count to assess whether other components and features of the blood were abnormal in *Pdlim7* mutant mice. We found slightly increased lymphocytes and monocytes in *Pdlim7* ^*-/-*^ (n=5; 4.00 ± 1.50 and 0.32 ± 0.11 x10^3^/µL, respectively) in comparison to WT (Table 3; n=5; 1.86 ± 0.63, p=0.0189 and 0.16 ± 0.05 x10^3^/µL, p=0.0472, respectively). However, all other parameters tested including red blood cells, hemoglobin, and platelets were similar between *Pdlim7* ^*-/-*^ (n=5) and WT (Table 3; n=5; p>0.05). 

**Table 3:** Blood chemistry panel and complete blood cell counts in adult *Pdlim7* ^*-/-*^ mice in comparison to WT controls.

**Parameter**	**Units**	**Pdlim7^+/+^**	**Pdlim7^-/-^**	**P-value**
Prothrombin time (PT)	sec	8.78 ± 0.58	8.62 ± 0.69	0.6416
Partial thromboplastin time (PTT)	sec	34.40 ± 0.67	34.52 ± 4.77	0.9632
Total protein	g/dL	4.78 ± 0.12	4.77 ± 0.18	0.8501
Albumin	g/dL	2.43 ± 0.44	2.68 ± 0.16	0.2213
Aspartate aminotransferase (AST)	IU/L	108.8 ± 40.39	96.83 ± 40.81	0.2298
Alanine aminotransferase (ALT)	IU/L	41.67 ± 10.56	50.67 ± 26.57	0.8031
Alkaline phosphatase	IU/L	77.50 ± 6.41	86.83 ± 16.87	0.2339
Gamma-glutamyl transpeptidase (GGT)	IU/L	1.00 ± 0	1.00 ± 0	NA
Total Bilirubin	mg/dL	0.12 ± 0.04	0.1 ± 0	0.6310
White blood cells	x 10^3^/µL	3.00 ± 0.87	4.44 ± 1.58	0.1118
Red blood cells	x 10^6^/µL	7.59 ± 0.82	7.94 ± 0.52	0.4361
Hemoglobin	g/dL	12.32 ± 0.78	11.92 ± 0.81	0.4493
Mean corpuscular hemoglobin	pg	15.24 ± 0.30	15.00 ± 0.12	0.1411
Red blood cell distribution width	%	35.52 ± 3.79	35.32 ± 6.75	0.9553
Platelets	x 10^3^/µL	550 ± 44.34	531 ± 76.01	0.6353
Lymphocytes	x 10^3^/µL	1.86 ± 0.63	4.00 ± 1.50	0.0189*
Monocytes	x 10^3^/µL	0.16 ± 0.05	0.32 ± 0.11	0.0472*
Granulocytes	x 10^3^/µL	0.18 ± 0.05	0.24 ± 0.09	0.3272

**Figure 6:**
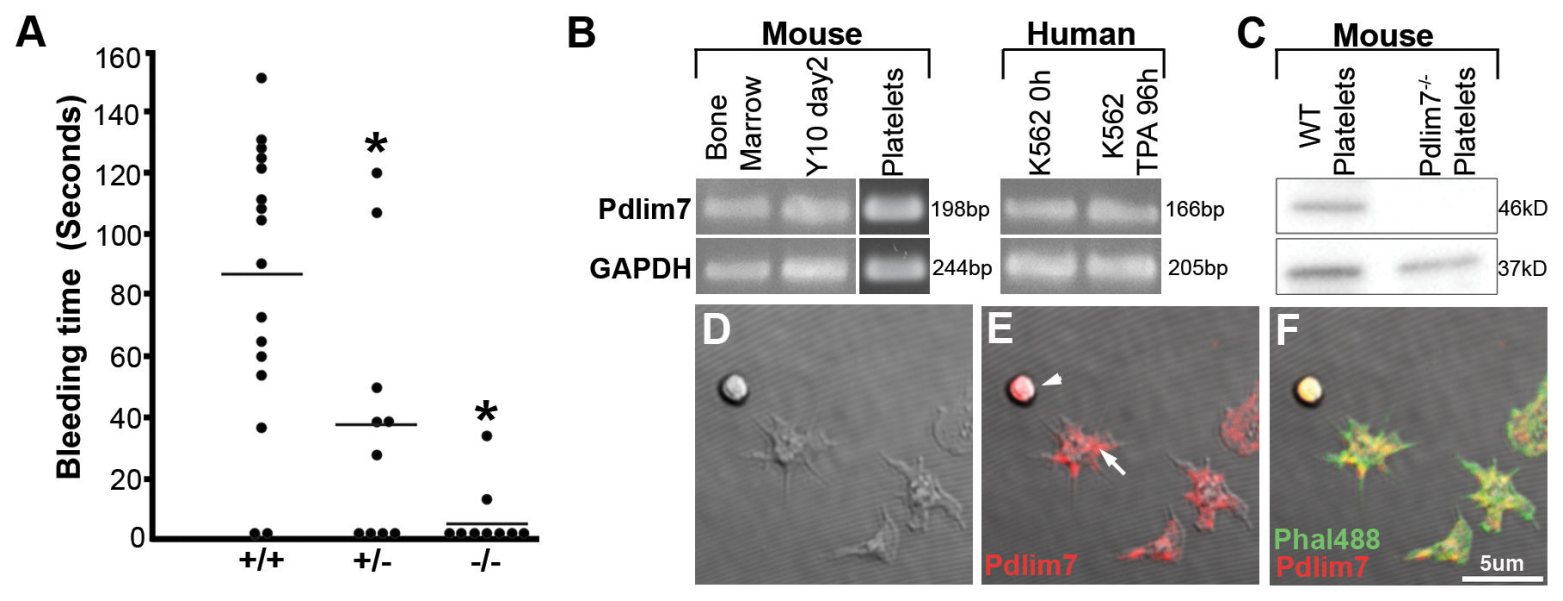
Pdlim7 is expressed in platelets and adult Pdlim7 deficient mice display hemostatic dysfunction. Upon tail clip, blood from 3-week old Pdlim7^-/-^ (n=9) mice clotted immediately compared to WT (n=16) controls with Pdlim7^+/-^ mice (n=10) exhibiting an intermediate phenotype (A). Horizontal line denotes mean bleed time for each genotype *p<0.01. Semi-quantitative RT-PCR identifies *Pdlim7* gene transcripts in WT mouse bone marrow, megakaryocyte cell line Y10 (undifferentiated, day2), and platelets as well as human bone marrow cell line K562 (undifferentiated, 0h; differentiated into proplatelets using TPA for 96h) using GAPDH as a control (B). Western blot analysis of platelets from WT mice demonstrates the presence of Pdlim7 proteins and the absence of Pdlim7 proteins in Pdlim7^-/-^ mice using GAPDH as a loading control (C). Confocal images of WT platelets spread on glass and immunostained with anti-Pdlim7 antibodies (red) and counterstained for F-actin (green) (D-F). Notably, Pdlim7 is distributed in resting (arrowhead) and spreading platelets (arrow) (E). Scale bar = 5µm.

To further evaluate the effect of Pdlim7 deficiency on hemostasis, we determined tail-bleeding times in normal and mutant mice (Figure 6A). The mean time to bleeding cessation was dramatically shorter in Pdlim7^-/-^ mice (n=9; 5.33 ± 11.7 s) in comparison to WT (n=16, 84.9 ± 45.9 s) with *Pdlim7* ^*+/-*^ mice exhibiting an intermediate phenotype (n=10, 38.3 ± 44.10 s, p=0.0007), suggesting a platelet dysfunction in Pdlim7-deficient mice. Next, we assessed Pdlim7’s expression in hematopoietic cells and detected Pdlim7 transcripts in murine and human bone marrow and megakaryocyte cell lines, the latter of which are the precursors to platelets (Figure 6B). In contrast to a previous report by Gupta et al. [11] that found no Pdlim7 (Enigma) mRNA expression in mouse platelets, using semi-quantitative RT-PCR, we could demonstrate Pdlim7 transcripts in WT murine platelets (Figure 6B). Additionally, we also demonstrate by Western-blot of platelet lysates that the RNA is translated into Pdlim7 proteins (Figure 6C). These findings are further supported by immunocytochemistry, revealing Pdlim7 co-localization with F-actin in resting and spreading platelets (Figure 6D-F). In context, these data reveal that despite approximately half of Pdlim7^-/-^ mice escaping premature death, the survivors also exhibit abnormal blood coagulation. The decreased bleeding time, but normal function of plasma coagulation factors, suggests that the prothrombotic phenotype in *Pdlim7* ^*-/-*^ mice is likely either platelet- or vascular smooth muscle-derived. These findings identify a novel and unexpected role for Pdlim7 in regulating mammalian hemostatic function. 

## Discussion

In the current study, we provide the first characterization of a global *Pdlim7* loss-of-function mouse. As Pdlim7 is widely distributed during embryogenesis, with robust expression in the heart, we hypothesized that a significant number of *Pdlim7* mutants die prematurely from a heart defect. However, we found that loss of Pdlim7 in mice does not cause detectable abnormalities in heart formation during embryogenesis. We demonstrate that Pdlim7 deficiency is associated with mild cardiac dysfunction and gross defects in hemostasis in both neonatal and adult mice, revealing a new and unexpected function for this protein. 

As a gene trap approach was used to generate the *Pdlim7* mutant mice, we cannot rule out the possibility that insertion of the Bgeo cassette into the genome has an effect on neighboring genes, leading to potential complications of interpretation of phenotypes. The fact that the intermediate phenotypes in body weight, bleeding time, and postnatal lethality correlate with the reduction of Pdlim7 transcript and protein levels in heterozygous mice, indicate that the demonstrated problems are the result of Pdlim7 loss-of-function. Considering the phenotypes in *Pdlim7* ^*+/-*^ mice, it appears that the significantly reduced Pdlim7 transcript and protein levels in the heterozygous mice leads to haploinsufficiency. 

### Pdlim7 is dispensable for heart valve development in mice, but is important for adult heart function

The apparent lack of molecular and cellular consequences of *Pdlim7* inactivation on valve development in mice are in stark contrast to the dramatic problems in valve formation in the two-chambered zebrafish heart following knockdown of *pdlim7* [14]. Recent live imaging studies of zebrafish cardiac development demonstrated that the AV valve develops through direct invagination of AV endocardial cells without a complete EMT [50,51] as in higher vertebrates and mammals [28,31,52]. Thus, one possible explanation is that certain Pdlim7-dependent steps related to valve development in zebrafish are not recapitulated in the four-chambered mouse heart. 

In mice, endocardial cells are considered to be the main contributors to mature AV valves [19]; therefore, we focused our attention on endocardially-derived valve defects in Pdlim7-deficient mice. Our investigation demonstrated that Pdlim7 is not required for AV cushion formation. However, we did find morphological aberrations in mature valves. Recent work by Wessels et al. revealed that in late fetal development, a subset of the AV valve leaflets largely consist of epicardial-derived cells (EPDCs) [53], shifting the long held paradigm that endocardial cells are the main contributors of valves. In both chicken [20] and mouse, we could detect Pdlim7-expressing cells in the epicardium, and it is therefore possible that Pdlim7 has a yet unknown function in EPDCs that supports valve remodeling. In light of these recent findings, future studies that would include EPDC lineage tracing to determine differences in the epicardial contribution to the AV valves following loss of Pdlim7 would be of great interest. However, given that Pdlim7-deficient adult mice also exhibit hemostatic dysfunction, we cannot rule out the possibility that the AV valve abnormalities are secondary to blood-related defects in these mice. 

Multi-species functional analyses of PDZ-LIM proteins are not well-documented; however, loss-of-function studies using the PDZ-LIM protein Ldb3 revealed less severe phenotypes and later onset in mice as compared to zebrafish and *Drosophila* [5,6,9]. In context with our Pdlim7 loss-of-function studies in zebrafish [3,14] and the mouse data presented here, an understanding is emerging that PDZ-LIM proteins are dispensable during mammalian organogenesis, but are rather important for organ function and homeostasis. The difference in presentation of the *Pdlim7* ^*-/-*^ phenotype between zebrafish and mouse may also lie in the fact that several PDZ-LIM proteins are expressed in overlapping domains in the mammalian heart, including Pdlim3 in the developing endocardial cushions, which would allow for other family members to compensate to some degree for the loss of Pdlim7 in our mutant model [4,5,8,54]. The possibility of functional redundancies of PDZ-LIM protein family members in the mouse complicate the analysis of single protein knockouts, and warrants further studies with PDZ-LIM protein double-mutant mice to uncover the full range of biological functions in the heart. 

### Loss of Pdlim7 in mice causes unique hypercoagulopathy

The systemic, pre-mortem thrombi observed in the non-surviving *Pdlim7* mutant mice and decreased tail bleed time in adult Pdlim7^-/-^ mice provides the first indication that Pdlim7 may function in regulating hemostasis. A predisposition to thrombosis has been reported to include three major factors: damage to the endothelium, abnormal blood flow, and/or aberrant hemostatic properties of the blood. We detected Pdlim7 proteins co-localized with F-actin in the vascular smooth muscle, but not the endothelium, suggesting that the hypercoagulopathy in *Pdlim7* mutant mice does not arise from primary defects in the endothelial layer. However, other PDZ-LIM proteins have been reported to function in the maintenance of muscle contractility [4,5,8,55], thus it is plausible that Pdlim7 could also function in smooth muscle contraction, and thereby influence blood flow. We report significant mortality of *Pdlim7* mutant mice shortly after birth. One possible explanation for this is that the stress endured by pups during labor and delivery causes an abnormal constriction of the blood vessels resulting in reduced blood flow, and triggering clot formation in the predisposed *Pdlim7* mutant mice. In a similar manner, smooth muscle contraction in the small muscular arteries of the tail could also explain the significantly decreased tail bleed time observed in adult Pdlim7^-/-^ mice. In the context of functional redundancy, it is worthwhile noting that, to our knowledge, Pdlim7 and Pdlim3 are the only PDZ-LIM family members with documented expression in mammalian smooth muscle [56], suggesting a lower likelihood of compensatory function in this muscle type. Future studies with Pdlim7^-/-^ bone marrow transplants into WT marrow-ablated mice will clarify whether there is a primary vessel issue or if a blood-specific defect has to be considered. 

Platelets are also a major player in coagulation. Upon vessel injury, they become activated and rapidly reorganize their actin cytoskeleton to adhere to the site of endothelial damage, triggering the formation of a fibrin-rich plug to prevent further blood loss [57,58]. The decreased bleeding time, but normal liver function and plasma coagulation factors in *Pdlim7* ^*-/-*^ survivors suggest that the etiology of the hypercoagulopathy may also be platelet-derived. Previous studies have shown that the PDZ-LIM protein Pdlim1 associates with actin stress fibers during shape change and spreading of human platelets [10]. A recent report by Gupta et al. revealed that lack of functional Pdlim1 in mice leads to marked hyperactivity of the collagen-glycoprotein VI (GPVI) signaling pathway in platelets, resulting in a prothrombotic phenotype [11]. While Gupta et al. found no other *Pdlim* genes expressed in murine platelets, we find *Pdlim7*-specific transcripts in developing and mature platelets, and demonstrate by Western-blot and immunocytochemistry that Pdlim7 proteins are dynamically distributed in platelets. Moreover, normal platelet counts in *Pdlim7* mutant mice indicate that platelet formation and survival are likely not affected, which supports the notion that the origin of the thrombi in *Pdlim7* mutant mice could be caused by abnormal platelet function. In light of Pdlim1’s role in platelet signaling, the analysis of platelet function in *Pdlim7* mutant mice is of great interest, and these investigations are currently ongoing in our laboratory. 

The global inactivation of the *Pdlim7* gene in the mouse presented an unexpected, but unique hypercoagulopathy phenotype. While Pdlim7 mutations in humans have not been reported, these new findings suggest that future studies investigating the clinical relevance of Pdlim7 may improve our understanding of the molecular basis of cardiac function and hemostasis in humans. 

## Supporting Information

Figure S1 ***Pdlim7*-deficient embryos do not reveal significant morphological defects during atrioventricular valve formation.** Embryos were sectioned in sagittal and transverse orientations and stained with Alcian blue/nuclear fast red. At E11.5, the developing AV cushions of Pdlim7^-/-^ (B; n=5) appeared relatively similar to WT controls (A; n=3). At E13.5 and E15.5, the differentiating AV valves of Pdlim7^-/-^ embryos (D; n=5 and F; n=5) were also similar to WT controls (n=4, C and n=3, E). Scale bar = 100µm (A-B) and 200µm (C-F). A = atrium; LV = left ventricle; MV = mitral valve; OFT = outflow tract; TV = tricuspid valve.
(TIF)

Figure S2 ***Pdlim7*^*-/-*^ embryos undergo endocardial EMT and exhibit a normal *Bmp2* expression pattern at the AV canal.** Atrioventricular cushion explants from WT (A-B; n=3) and *Pdlim7* ^*-/-*^ (C-D; n=3) embryos cultured for 48 hours on collagen gels. Mesenchymal cells visualized at the gel surface (0µm, A, C) and at a depth of 40µm into the gel (B, D, arrows) did not show differences in distribution. Whole-mount in situ hybridization of *Bmp2* at E9.5 and E10.5 in WT (E, G; n=5 and 4, respectively) and *Pdlim7* ^*-/-*^ embryos (F, H; n=3 and 4, respectively) demonstrates similar expression patterns in *Pdlim7* mutant hearts. qRT-PCR array analysis of AV canals of E10.5 *Pdlim7* ^*-/-*^ embryos reveals normal expression of several genes important for endocardial EMT compared to WT controls (I). A = atrium, LV = left ventricle, Myo = myocardium.
(TIF)

Figure S3 **Adult *Pdlim7*^*-/-*^ mice exhibit blood clots attached to the atrioventricular valves.** In some cases, prior to perfusion, blood clots were observed attached to the mitral valve in 3-month old *Pdlim7* ^*-/-*^ adult mice (asterisk and arrowhead, B-B’), but not in WT littermates (A). Scale bar = 200 µm (A-B), 20 µm (B’).
(TIF)

Video S1 **Three-dimensional reconstructions of adult WT and *Pdlim7*^*-/-*^ atrioventricular valves.** Serial sections of adult hearts stained with either Masson’s trichrome or Alcian blue to distinguish valve tissue were aligned to extract the 3D voxels used to compute a 3D reconstruction of the cardiac valves.
(MOV)

Materials S1 **Description of the materials and methods for the supporting information. **
(PDF)
