# Supplementary figures and images for: Loss of the Cytoskeletal Protein Pdlim7 Predisposes Mice to Heart Defects and Hemostatic Dysfunction
Source: PLoS One. 2013 Nov 20;8(11):e80809. doi: 10.1371/journal.pone.0080809 (PMC3835322; doi:10.1371/journal.pone.0080809)

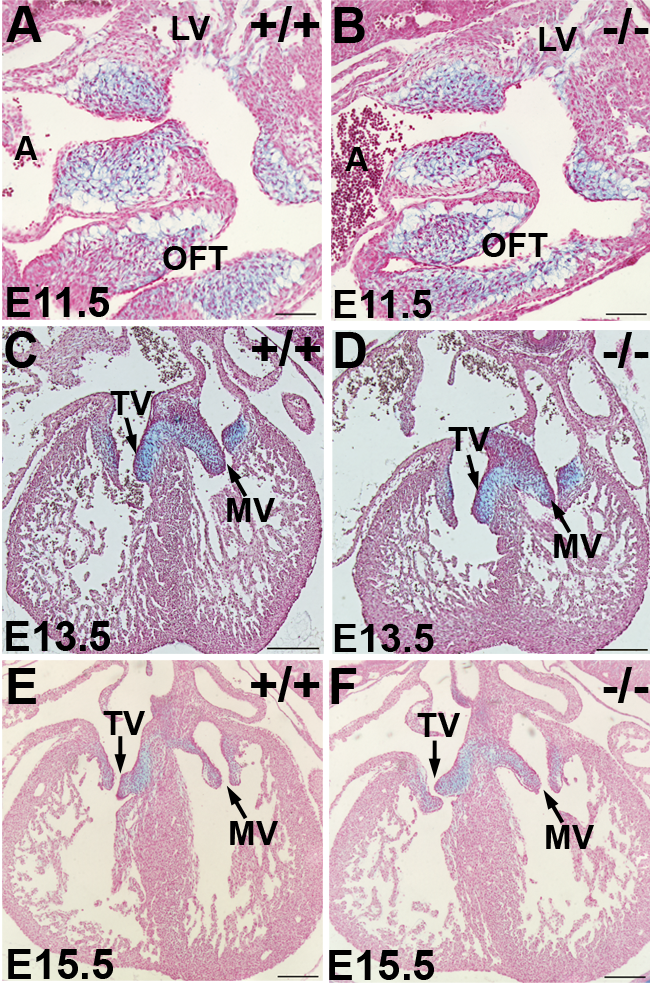

Supplement: Figure S1 — Pdlim7-deficient embryos do not reveal significant morphological defects during atrioventricular valve formation. Embryos were sectioned in sagittal and transverse orientations and stained with Alcian blue/nuclear fast red. At E11.5, the developing AV cushions of Pdlim7-/- (B; n=5) appeared relatively similar to WT controls (A; n=3). At E13.5 and E15.5, the differentiating AV valves of Pdlim7-/- embryos (D; n=5 and F; n=5) were also similar to WT controls (n=4, C and n=3, E). Scale bar = 100µm (A-B) and 200µm (C-F). A = atrium; LV = left ventricle; MV = mitral valve; OFT = outflow tract; TV = tricuspid valve. (TIF) [file pone.0080809.s001.tif]

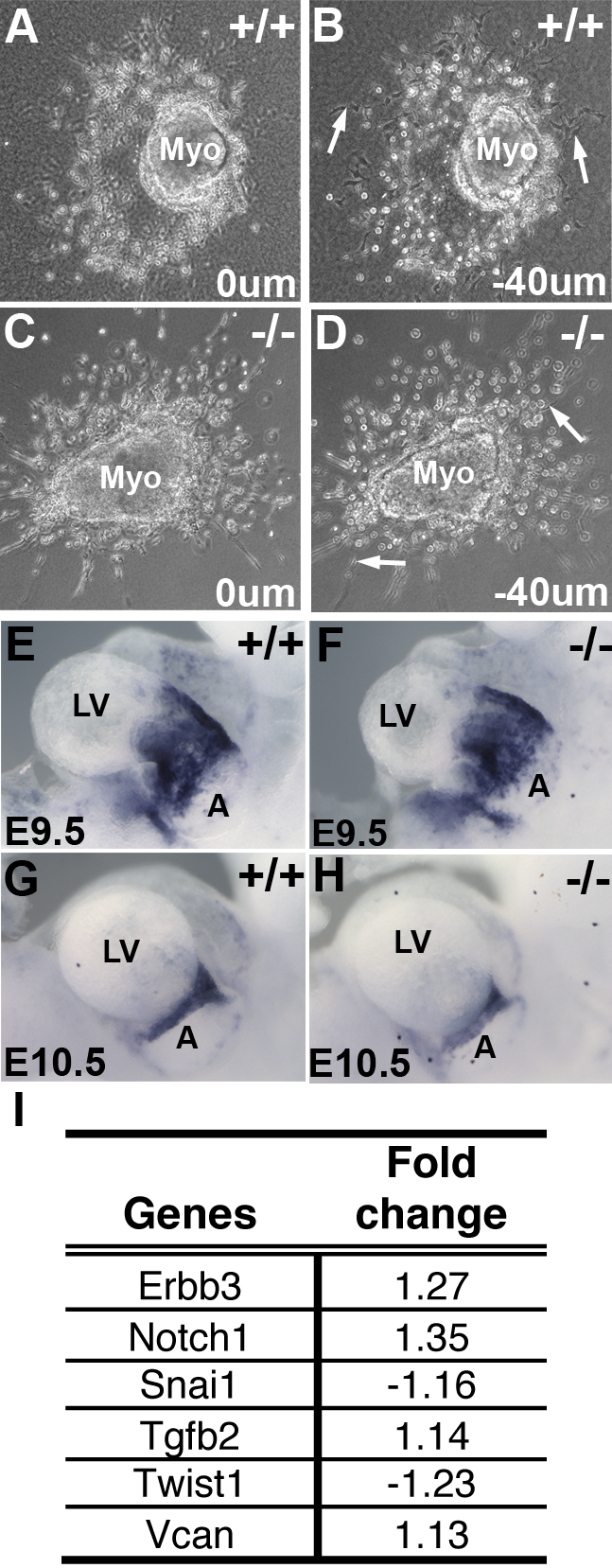

Supplement: Figure S2 — Pdlim7-/- embryos undergo endocardial EMT and exhibit a normal Bmp2 expression pattern at the AV canal. Atrioventricular cushion explants from WT (A-B; n=3) and Pdlim7 -/- (C-D; n=3) embryos cultured for 48 hours on collagen gels. Mesenchymal cells visualized at the gel surface (0µm, A, C) and at a depth of 40µm into the gel (B, D, arrows) did not show differences in distribution. Whole-mount in situ hybridization of Bmp2 at E9.5 and E10.5 in WT (E, G; n=5 and 4, respectively) and Pdlim7 -/- embryos (F, H; n=3 and 4, respectively) demonstrates similar expression patterns in Pdlim7 mutant hearts. qRT-PCR array analysis of AV canals of E10.5 Pdlim7 -/- embryos reveals normal expression of several genes important for endocardial EMT compared to WT controls (I). A = atrium, LV = left ventricle, Myo = myocardium. (TIF) [file pone.0080809.s002.tif]

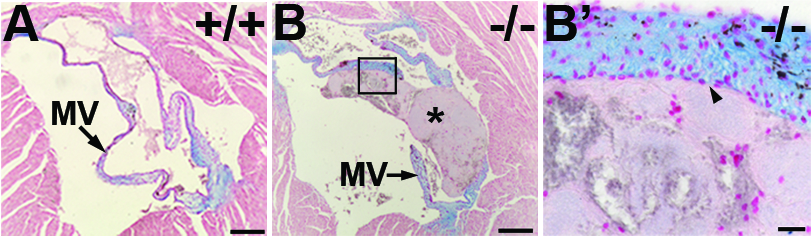

Supplement: Figure S3 — Adult Pdlim7-/- mice exhibit blood clots attached to the atrioventricular valves. In some cases, prior to perfusion, blood clots were observed attached to the mitral valve in 3-month old Pdlim7 -/- adult mice (asterisk and arrowhead, B-B’), but not in WT littermates (A). Scale bar = 200 µm (A-B), 20 µm (B’). (TIF) [file pone.0080809.s003.tif]
