# Supplementary material for: Loss of the Cytoskeletal Protein Pdlim7 Predisposes Mice to Heart Defects and Hemostatic Dysfunction
Source: PLoS One. 2013 Nov 20;8(11):e80809. doi: 10.1371/journal.pone.0080809 (PMC3835322; doi:10.1371/journal.pone.0080809)
Supplement: Materials S1 — Description of the materials and methods for the supporting information. (PDF) [file pone.0080809.s005.pdf]

Supplemental Material and Methods

Mouse atrioventricular cushion explants

Mouse atrioventricular (AV) cushion mesenchymal cell transformation assay was performed as previously described [1]. In short, E9.25 (16-18 somite) *Pdlim7*$^{-/-}$ and WT embryos were harvested into Earl's balanced salt solution and the AV canal removed and cut longitudinally to expose the lumen. The tissue was placed luminal side down onto the surface of a drained collagen (1mg/ml) gel and incubated at 37°C in a humid CO_2 incubator for 2 hours. After 2 hours, complete OptiMEM (1% heat inactivated Fetal bovine serum, 5μg/mL insulin, 5μL/mL transferrin, 5ng/mL selenium, 100units/mL penicillin, and 100mg/mL streptomycin) was added to the sample. After 48 hours, explants were imaged on a Leica DM-IRB Inverted microscope equipped with a QImaging Retiga 4000R camera using OpenLab (Improvision) software.

In situ hybridization

Whole mount *in situ* hybridization was performed as previously described [2] using an Insitu Pro VSi robot (Intavis). The Bmp2 probe was a kind gift from Dr. Jim Martin. Embryos were imaged on a Leica MZ16 stereomicroscope fitted with a Leica DFC490 color camera using ImagePro MC (MediaCybernetics) software.

PCR array

The RT^2 Profiler Mouse Epithelial-to-Mesenchymal Transition PCR Array (PAMM-090Z; SABiosciences) was performed according to manufacturer guidelines on the AV canals

of E10.5 *Pdlim7*$^{-/-}$ and WT embryos. The AV canals from at least 3 litters were polled together to obtain enough RNA. The qPCR reactions were carried out using a BioRad iQ5 thermocycler and data analyzed using a web-based software program provided by the manufacturer.

Supplemental References

1. Sugi Y, Yamamura H, Okagawa H, Markwald RR (2004) Bone morphogenetic protein-2 can mediate myocardial regulation of atrioventricular cushion mesenchymal cell formation in mice. Dev Biol 269: 505-518.
2. Wirrig EE, Snarr BS, Chintalapudi MR, O'Neal J L, Phelps AL, et al. (2007) Cartilage link protein 1 (Crtl1), an extracellular matrix component playing an important role in heart development. Dev Biol 310: 291-303.

Table S1. *Pdlim7* gene expression in E18.5 embryos.

Tissue	Expression level
Aorta	+++
Alveoli (right and left lungs)	++
Bladder wall	+++
Bronchus	+
Cardiac endocardium	++
Coronary vessels	++
Costal cartilage	++
Diaphragm	++
Ductus venosus	+
Duodenum	+++
Esophagus	+++
Hepatic venous plexus	+
Inferior vena cava	+
Intercostal muscles	+++
Kidney	+
Mitral valve attachment point	++
Pulmonary artery	++
Rectum	+++
Stomach	+++
Trachea	++
Tricuspid valve attachment point	++
Umbilical vessels	+++
Urethra	++

Table S2. Echocardiography measurements of 3-month old *Pdlim7*$^{-/-}$ mice in comparison to WT controls.

	**Parasternal long axis**			**Short axis**		
Measurement	Pdlim7$^{+/+}$	Pdlim7$^{-/-}$	P-value	Pdlim7$^{+/+}$	Pdlim7$^{-/-}$	P-value
LVID;d	3.88 ± 0.17	3.86 ± 0.10	0.6799	3.49 ± 0.38	3.75 ± 0.22	0.0975
LVID;s	2.69 ± 0.28	2.70 ± 0.30	0.9522	2.35 ± 0.37	2.60 ± 0.27	0.1261
LVPW;d	0.82 ± 0.10	0.79 ± 0.10	0.6498	0.78 ± 0.20	0.79 ± 0.07	0.8290
LVPW;s	1.17 ± 0.16	1.08 ± 0.22	0.3586	1.08 ± 0.20	1.07 ± 0.12	0.9674
LVAW;d	0.83 ± 0.08	0.74 ± 0.07	0.0261	0.81 ± 0.07	0.77 ± 0.10	0.3092
LVAW;s	1.24 ± 0.17	1.11 ± 0.17	0.1166	1.16 ± 0.15	1.12 ± 0.11	0.5199
%FS	30.7 ± 5.68	30.6 ± 6.63	0.9856	32.8 ± 5.77	30.1 ± 5.59	0.3339
%EF	50.5 ± 8.70	49.5 ± 8.89	0.8218	NA	NA	NA

LVID = left ventricular inner dimension; LVPW = left ventricular posterior wall; LVAW = left ventricular anterior wall; d = diastole; s = systole. Values are in millimeters unless otherwise noted.

Table S3. Tei index measurements in 3-month old *Pdlim7*$^{-/-}$ mice in comparison to WT controls.

	**Apical 4-chamber view**		
Measurement	Pdlim7$^{+/+}$	Pdlim7$^{-/-}$	P-value
IVCT (ms)	9.64 ± 1.82	13.8 ± 2.02	0.0005
ET (ms)	49.3 ± 5.53	48.8 ± 3.33	0.6650
IVRT (ms)	16.2 ± 1.72	18.3 ± 3.53	0.1558

IVCT = isovolumetric contraction time; ET = ejection time; IVRT = isovolumetric relaxation time.
